# Diversity and function of culturable actinobacteria in the root-associated of *Salvia miltiorrhiza* Bunge

**DOI:** 10.7717/peerj.11749

**Published:** 2021-07-09

**Authors:** Yu-Rui Wu, Cui-Bai Li, Yan-Hong Wu, Lan Li, Bo Li, Wen-Bo Li, Bu-Jin Ma, Zhu-Yun Yan

**Affiliations:** School of Pharmacy, Chengdu University of Traditional Chinese Medicine, State Key Laboratory of Characteristic Chinese Medicine Resources in Southwest China, Chengdu, China

**Keywords:** *Salvia miltiorrhiza* Bunge, Root-associated, Actinobacteria, Metagenomic, Function

## Abstract

The root-associated actinobacteria play important roles in plant growth, nutrient use, and disease resistance due to their functional diversity. *Salvia miltiorrhiza* is a critical medicinal plant in China. The root actinobacterial community structure has been studied; however, the functions of root-associated actinobacteria of *S. miltiorrhiza* have not been elucidated. This study aimed to decipher the diversity and function of the culturable root-associated actinobacteria in plant growth using culture-dependent technology and culturable microbe metagenomes. We isolated 369 strains from the root-associated actinobacteria, belonging to four genera, among which *Streptomyces* was dominant. Besides, the functional prediction revealed some pathways related to plant growth, nitrogen and phosphorus metabolism, and antagonistic pathogens. We systematically described the diversity and functions of the culturable root-associated actinobacteria community. Our results demonstrated that the culturable root-associated actinobacteria of* S. miltiorrhiza* have rich functionalities, explaining the possible contribution of culturable root-associated actinobacteria to *S. miltiorrhiza*’s growth and development. This study provides new insights into understanding the function of the culturable root-associated actinobacteria and can be used as a knowledge base for plant growth promoters and biological control agent development in agriculture.

## Introduction

The root system is a complex ecosystem inhabited by diverse and active microorganisms. To date, it is known that root-associated microbiomes (Edwards et al., 2015), especially actinobacteria, have beneficial effects on plant growth and development. Actinobacteria are Gram-positive bacteria with high G+C content, isolated from different habitats (Chen et al., 2019), such as root, rhizosphere, and soil. These taxa can produce various biologically active compounds, especially antibiotics, and have antagonistic pathological characteristics. Therefore, they are extensively used in agricultural industries (Sousa & Olivares, 2016; Vurukonda, Giovanardi & Stefani, 2018). It has been demonstrated that actinobacteria protect crops against pathogens, including tomato (Goudjal et al., 2016), pepper (Liotti, da Silva Figueiredo & Soares, 2019), cereal (Newitt et al., 2019), rice (Xu et al., 2017), and strawberry (Marian et al., 2020).

Evidence has shown that the root-associated actinobacteria contribute to plant growth promotion (Dutta & Thakur, 2020), and plant stress alleviation (Esmaeil Zade, Sadeghi & Moradi, 2018; Selim et al., 2019), through diverse mechanisms such as indole-3-acetic acid (IAA) biosynthesis, siderophore production, and phosphate solubilization. Studies have investigated the impact of actinobacteria on plant growth promotion and seed germination rate improvement (Zamoum et al., 2015; Allali et al., 2019; Borah & Thakur, 2020). Also, actinobacteria ability to mitigate the negative impact of abiotic stress on the growth and physiology of crops, such as wheat (Yandigeri et al., 2012; Li et al., 2019), maize (Warrad et al., 2020), tomato (Abbasi, Sadeghi & Safaie, 2020) has been investigated. However, most knowledge on the diversity and function of the root-associated actinobacteria is derived from common crops. To date, most research focuses on characterizing medicinal plants’ community composition and evaluating antibiotics production (Quinn et al., 2020) while only a few studies have examined host-related functions.

Culture-dependent technology is the most classic method to study the microbe. In fact, many microorganisms are unculturable for various reasons (Pham & Kim, 2012). However, for agriculture, it is important to focus on microorganisms with practical utility, and the key is culture-dependent technology. Subsequently, with the emergence of metagenomics, this technology has been used to identify different microbial genes and their functionalities, allowing the understanding of a global view of the microbial community (Tian, Cao & Zhang, 2015). Hence, combining culture-dependent and metagenomic technologies allows us to map the functions of culturable microorganisms fully. This map will help us understand the role of microorganisms in the interaction between plants and microorganisms.

In China, *S. miltiorrhiza* is a medicinal plant used to treat cardiovascular and cerebrovascular diseases (Jung et al., 2020), in which the root actinobacteria have previously been reported (Li et al., 2010; Yan et al., 2016), but until now, only the *Streptomyces* have been isolated. Besides, no study has assessed the function of the root-associated actinobacteria of this species. Given that *S. miltiorrhiza* is one of the most important medicinal plants, elucidating the structure and function of the culturable root-associated actinobacteria on its growth and health is inevitable.

This study used culture-dependent technology and metagenomics to explore the diversity and function of the culturable root-associated actinobacterial communities in *S. miltiorrhiza* systematically. To our knowledge, this is the first report on the functional analysis of the culturable root-associated actinobacteria of *S. miltiorrhiza*. This study provides new insights into understanding the function of the culturable root-associated actinobacteria and provides a basis to further study the application of the culturable root-associated actinobacteria of *S. miltiorrhiza*.

## Material & Methods

### Sample collection

Samples used in this study were collected from Sichuan Province (E: 104.54°, N: 30.94°), China, on March 20th, 2019. The samples were composed of roots and rhizosphere soil. During sampling, the roots of healthy plants were excavated, the clumpy soil on the roots’ surfaces was discarded. Soil particles closely adhering to the surface of the roots were collected as rhizosphere soil. The roots and rhizosphere soil were placed in sterile paper bags and brought into the laboratory at 4 °C. Endophytic and rhizosphere actinobacteria were isolated within 48 h.

### Surface sterilization and isolation actinobacteria

The root surfaces sterilization protocol included three steps: the tissue surface was immersed in 1% (v/v) sodium hypochlorite for 3 min; soaked in 75% (v/v) ethanol for 30 s and finally rinsed five times with sterile distilled water. The surface sterilization effectiveness was evaluated by plating the final wash water on Gause’s No. l medium. The sterilized surface samples were cut into small fragments and homogenates. A little sterile distilled water was added and mixed, then 0.2 mL suspension was dispersed onto six actinobacteria-selective mediums: (A) Gause’s No. l medium (Zhang & Zhang, 2011); (B) Gauze’s agar medium No.2, modified (Yan et al., 2016); (C) HVA (Qin et al., 2009); (D) Trehalose-proline agar (Qin et al., 2009); (E) TWYE medium (Qin et al., 2009); and (F) S medium (Cao et al., 2004).

For rhizosphere soil samples, 10 g of soil was added to 90 mL of sterile water and shaken at 100 × g at 4 °C for 3 min. A 10^−2^ soil diluent was prepared using the 10-fold serial dilution method. Then, the homogenization buffer or diluent was placed in the media (A), (C), and (G). All media were supplemented with potassium dichromate (K_2_Cr_2_O_7_) (75 mg L^−1^) and penicillin sodium (2 mg L^−1^) and incubated at 28 °C for four weeks before being monitored every seven days for microbial growth. Colonies were observed and selected according to their characteristics and morphology. The purified actinobacterial isolates were stored on the Gause’s No. l medium at 4 °C and in 30% glycerol at −80 °C.

### Isolates Identification

For each isolate, genomic DNA was extracted using the HiPure Bacterial DNA Kit per to the manufacturer’s instructions. The 16S rRNA gene was amplified using universal primers 27F (5′-CAGAGTTTGATCCTGGCT-3′) and 1492R (5′-AGGAGGTGATCCAGCCGCA-3′). The PCR mixture (25 µL) contained 22 µL 1 ×Taq PCR Master Mix, 1 µL of the DNA template, and 1 µL of each primer. We used the following amplification protocol: PCR was initialized by a denaturing step at 94 °C for 4 min followed by 34 amplification cycles with denaturation at 94 °C for 30 s, annealing at 55 °C for 30 s, extension 72 °C for 1min, and a final polymerization step at 72 °C for 10 min. PCR products were stored at 4 °C and sequenced by Beijing TSINGKE Biological Technology. The obtained forward and reverse DNA sequence reads were used to produce contig sequence using DNASTAR Lasergene v.7.1 software (Burland, 2000). The 16S rRNA gene sequences were compared with those of NCBI. A maximum likelihood phylogenetic tree of 16S rRNA gene sequences was constructed using the molecular evolutionary genetics analysis (MEGA) software, v.7.0 (Kumar, Stecher & Tamura, 2016).

### Analysis of the actinobacteria root-associated function

For function analysis, metagenomics was performed on the DNA mixture of the root-associated actinobacteria. DNA was mixed at 50 ng/L per strain, and three replicates were set. The purity and integrity of DNA were analysed by agarose gel electrophoresis (AGE); then, the library was constructed. After library quantification, Illumina PE150 sequencing was performed. Preprocessing of the raw data obtained from the Illumina HiSeq sequencing platform using Readfq (v.8, https://github.com/cjfields/readfq) was conducted to acquire clean data for subsequent analysis. The clean data were assembled and analysed using SOAPdenovo software (Qin et al., 2010; Karlsson et al., 2012; Karlsson et al., 2013) (v.2.04, https://sourceforge.net/projects/soapdenovo2/). The scaftigs predicted the open reading frame (ORF) using the MetaGeneMark (v.2.10, http://topaz.gatech.edu/GeneMark/) software (Zhu, Lomsadze & Borodovsky, 2010; Karlsson et al., 2012; Karlsson et al., 2013; Mende et al., 2012; Nielsen et al., 2014; Oh et al., 2014). For ORF prediction, the CD-HIT software (Li & Godzik, 2006; Fu et al., 2012) (v.4.5.8, http://www.bioinformatics.org/cd-hit) was adopted for redundancy and to obtain a unique initial gene catalogue. The DIAMOND software (Buchfink, Xie & Huson, 2015) (v0.9.9.110, https://github.com/bbuchfink/diamond/) was used to blast Unigenes to the functional database, including the Kyoto Encyclopedia of Genes and Genomes (KEGG) database (Kanehisa et al., 2006; Kanehisa et al., 2014) (v.2018-01-01, http://www.kegg.jp/kegg/), Evolutionary Genealogy of Genes: Non-supervised Orthologous Groups (eggNOG) database (Powell et al., 2013) (v.4.5, http://eggnogdb.embl.de/#/app/home), and Carbohydrate-Active enZYmes (CAZy) database (Cantarel et al., 2009) (v.201801, http://www.cazy.org/) for comparison, function annotation and abundance analysis.

### Screening for antimicrobial activity

Antimicrobial assays of 72 actinobacterial strains against the bacterial and fungal strains were performed using the agar diffusion method (Nimaichand et al., 2015). Six microorganisms, including two Gram-positive bacteria: *Staphylococcus aureus*, *Bacillus cereus*; two Gram-negative bacteria: *Escherichia coli* and *Pseudomonas aeruginosa*; and a yeast, *Candida albicans*, the *S. miltiorrhiza* pathogen, *Fusarium oxysporum*, served as the test microorganisms. The test bacteria and fungi were activated on LB broth and PDB for 12 h and 2 d for 30 °C, respectively. They were adjusted to approximately 10^8^ and 10^6^ CFU/mL with sterile distilled water to prepare a microbial and spore suspension. A one mL bacterial/fungal solution was then added to 100 mL LB/PDA media, mixed gently, and then slowly poured on the Petri dish (90 mm), which served as the test plate. The actinobacterial disks (nine mm) were cultured on Gause’s No. l medium for seven days at 28 °C. They were transferred to media seeded with the test bacteria and fungi in triplicate and were incubated at 37 °C for 24 h and 28 °C for 2 d, respectively. The inhibition zone (mm) was observed and documented. All experiments were replicated three times.

### Screening for plant growth-promoting activity

Seventy-two strains were tested for plant growth-promoting activities in vitro. Siderophore production was studied by the method described by Alexander & Zuberer (1991). Briefly, using chrome azurol agar (CAS), colonies that exhibited a yellow halo after proliferation were classified as positive for siderophore production. The strains’ phosphate solubilization capability was analyzed on Pikovskaya (PVK) medium (Gupta et al., 1994). Finally, the phosphate solubilization capacity was determined by a halo surrounding colonies on Pikovskaya medium. IAA production was checked using Salkowski reagent in a tryptophan-amended medium (Gordon & Weber, 1951). The development of pink coloration indicates IAA production. Optical density was taken at 530 nm, and compared to a standard curve of indole-3-acetic acid of 5 µg/mL to 100 µg/mL at 530 nm. Nitrogen fixation activity was tested using nitrogen-free Ashby media (Li et al., 2008). Strain growth proved to have the ability to fix nitrogen. All strains were incubated for seven days and replicated three times.

## Results

### Isolation of the root-associated actinobacteria

**Figure 1:**
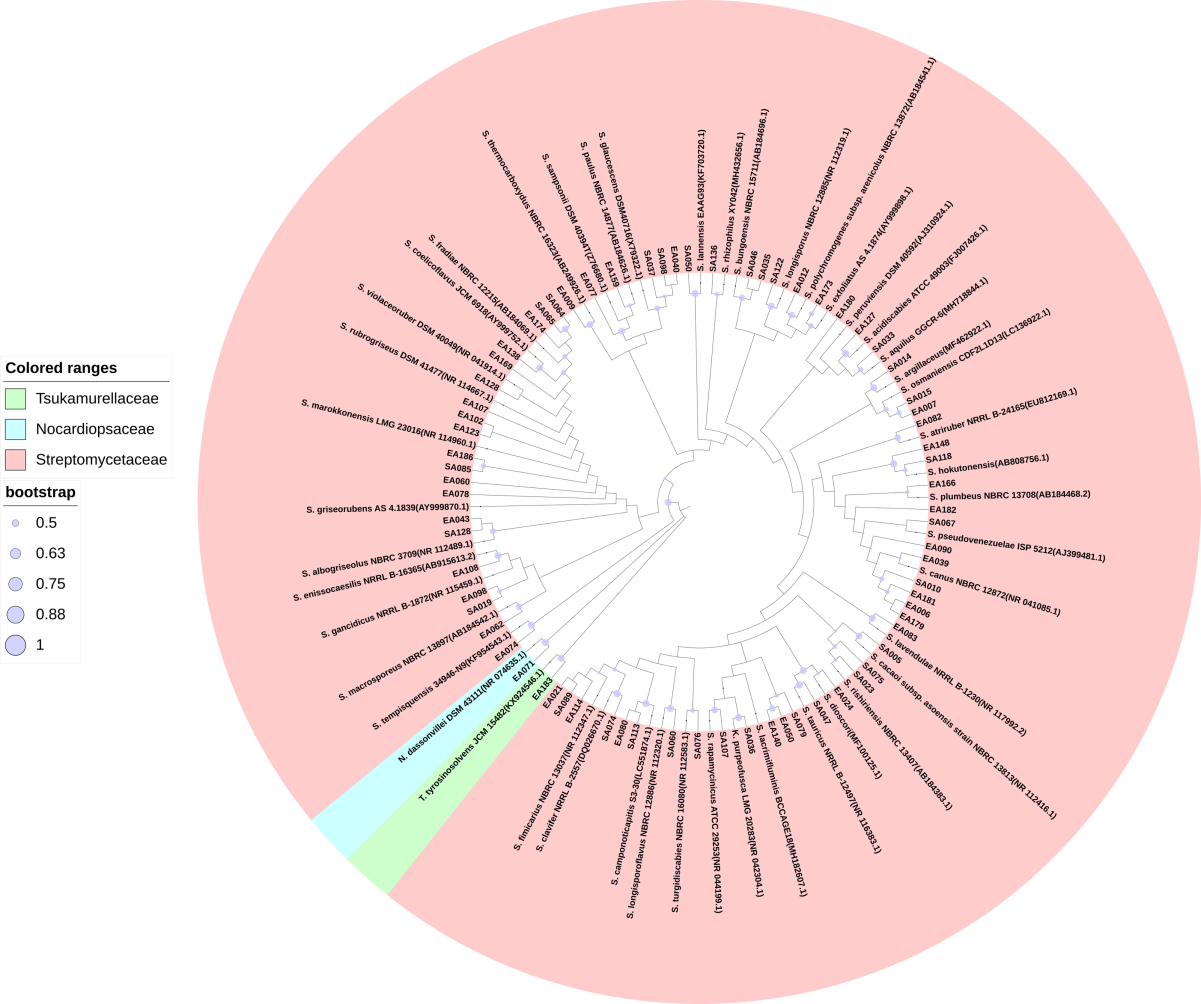
Maximum Likelihood phylogenetic tree based on 16S rRNA gene of the actinobacterial isolates. Maximum Likelihood phylogenetic tree based on 16S rRNA gene of the actinobacterial isolates and closely related actinobacterial type strains shown that the isolates were clustered within three families, four genera, and 46 species. The purple circle of the nodes indicates bootstrap values based on 1,000 replicates.

We isolated 201 endophytic actinobacteria and 168 rhizosphere soil actinobacteria from the root of *S. miltiorrhiza* and its rhizosphere soil. These organisms’ identities were determined based on 16S rRNA gene sequence analysis. The sequences of 100% identity were clustered into one species. According to the analysis of sequencing results, many isolated strains had the same 16S rRNA sequence. We obtained 72 actinobacterial strains after merging the duplicated sequences (GenBank accession numbers MW642091 to MW642162). Based on the BLAST result and phylogenetic tree analysis, actinobacteria obtained in this study were identified and categorized into three families (Streptomycetaceae, Tsukamurellaceae, and Nocardiopsaceae), four genera (*Streptomyces*, *Nocardiopsis*, *Tsukamurella*, and *Kitasatospora*), and 46 species, among which *Streptomyces* was the dominant genus. In the 46 species of actinobacterial, 14 were unique to rhizosphere soil, and 15 were unique to the root compartment. Most of the strains exhibited more than 98.65% similarity to other published type species. Table S1 presents the percentages of 16S rRNA gene sequence similarities (98.22%–100%) of these isolates to the closest type strains. Figure 1 shows the phylogenetic tree.

### Function analysis of the root-associated actinobacteria

We explored the root-associated actinobacterial functionalities using Illumina metagenome sequencing, focusing on the functional genes, enzymes, and pathways involved in PGP activities. The metagenomic contigs were annotated in eggNOG, CAZy, and KEGG databases. We found that the root-associated actinobacteria had potent functions.

**Figure 2:**
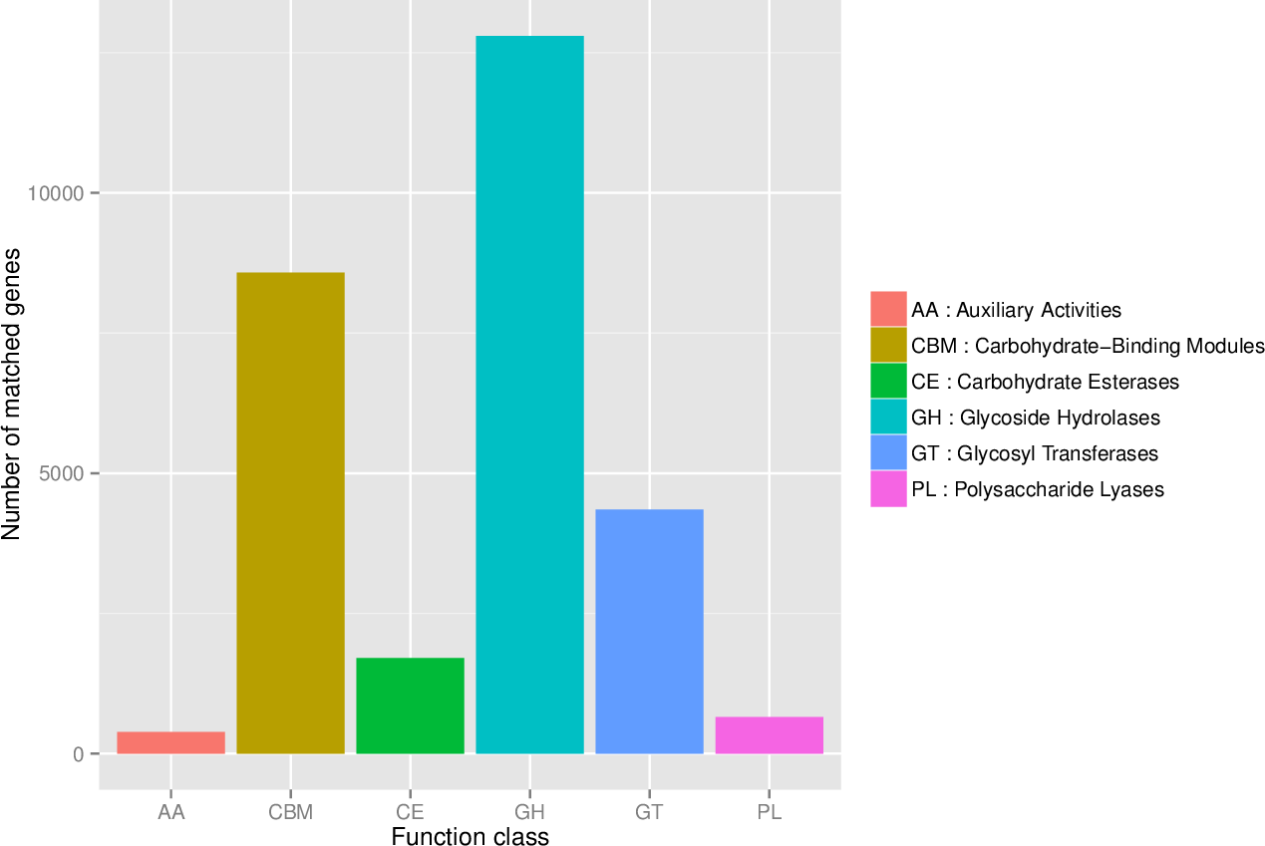
The number of genes annotated at CAZy database level 1.

**Figure 3:**
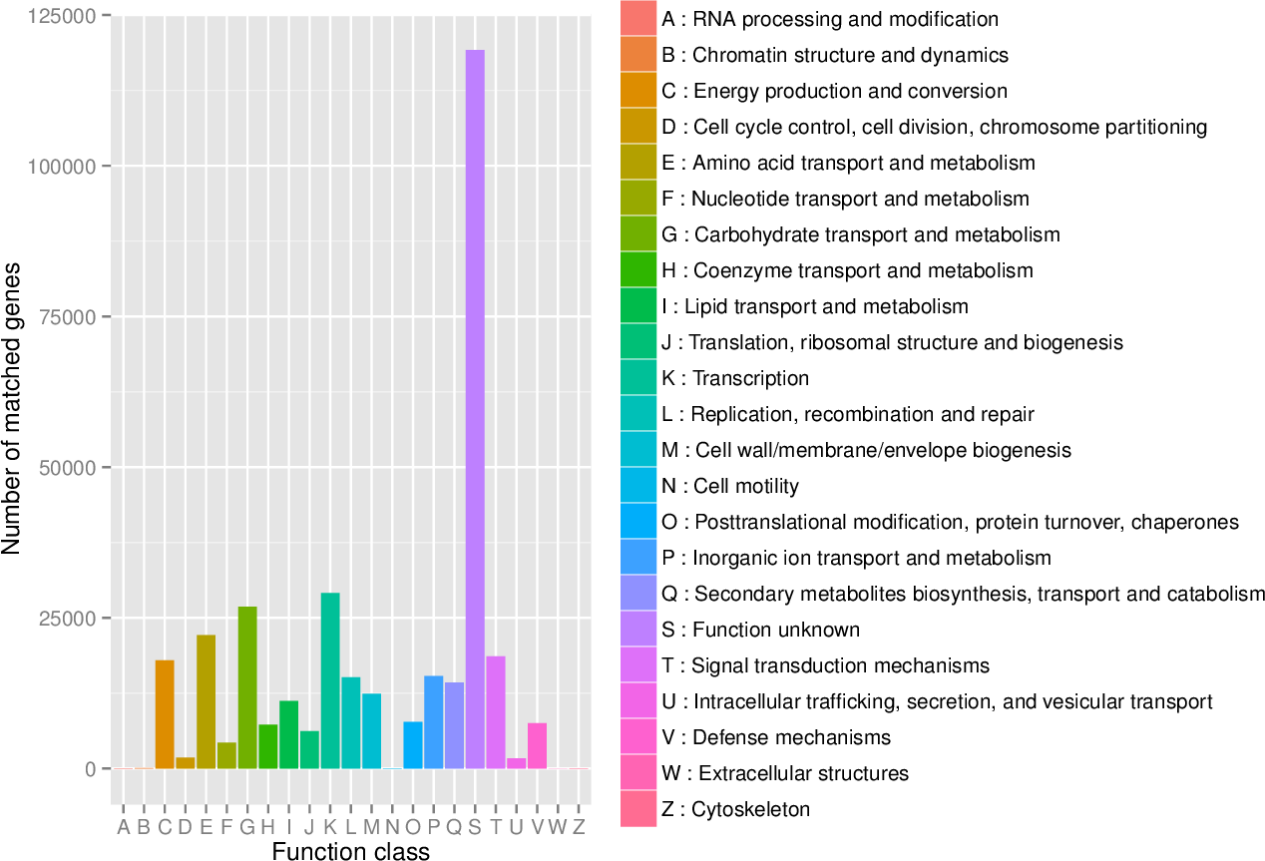
The distribution of genes in the eggNOG functional annotation at level 1.

First, in our study, annotation using KEGG database showed that the genes involved in Metabolic was the most predominant. There were 71 maps whose relative abundance was more than 0.1% in Metabolism, among which Carbohydrate metabolism accounts for the largest proportion, followed by Amino acid metabolism (Table S2). Second, through the analysis of CAZy reference sequences, we observed that Glycoside Hydrolases and Glycoside Hydrolases were the dominant CAZy families, followed by Glycoside Hydrolases, Carbohydrate Esterases, Polysaccharide Lyases, and Auxiliary Activities (Fig. 2, Table S3). Third, the functional annotations by eggNOG process categories indicated Transcription [K]was the dominant function among 23 categories, followed by Carbohydrate transport and Metabolism [G] (>25000), Amino acid transport and metabolism [E], Signal transduction mechanisms[T], and Energy production and conversion[C] (>17000). The lowest number of genes is assigned to Cell motility[N], Chromatin structure and dynamics[B], RNA processing and modification[A], Extracellular structures[W], and Cytoskeleton[Z] (Fig. 3, Table S4).

**Figure 4:**
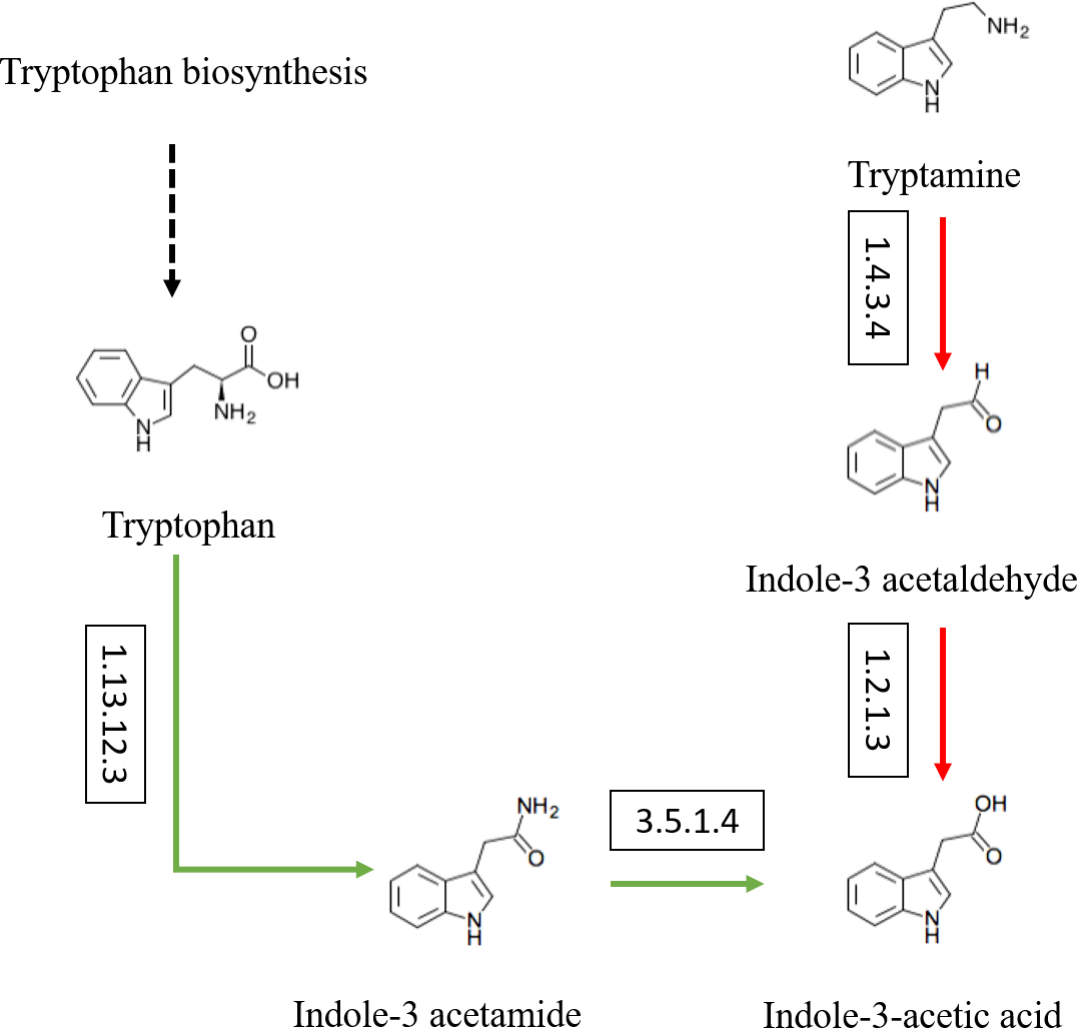
Actinobacteria from the root-associated of *S. miltiorrhiza* are involved in the pathway of IAA (indole-3-acetic acid) biosynthesis. Arrows indicate the IAM pathway in green and the TAM pathway in red. Numbers inside boxes represent EC numbers of the enzymatic reaction.

**Figure 5:**
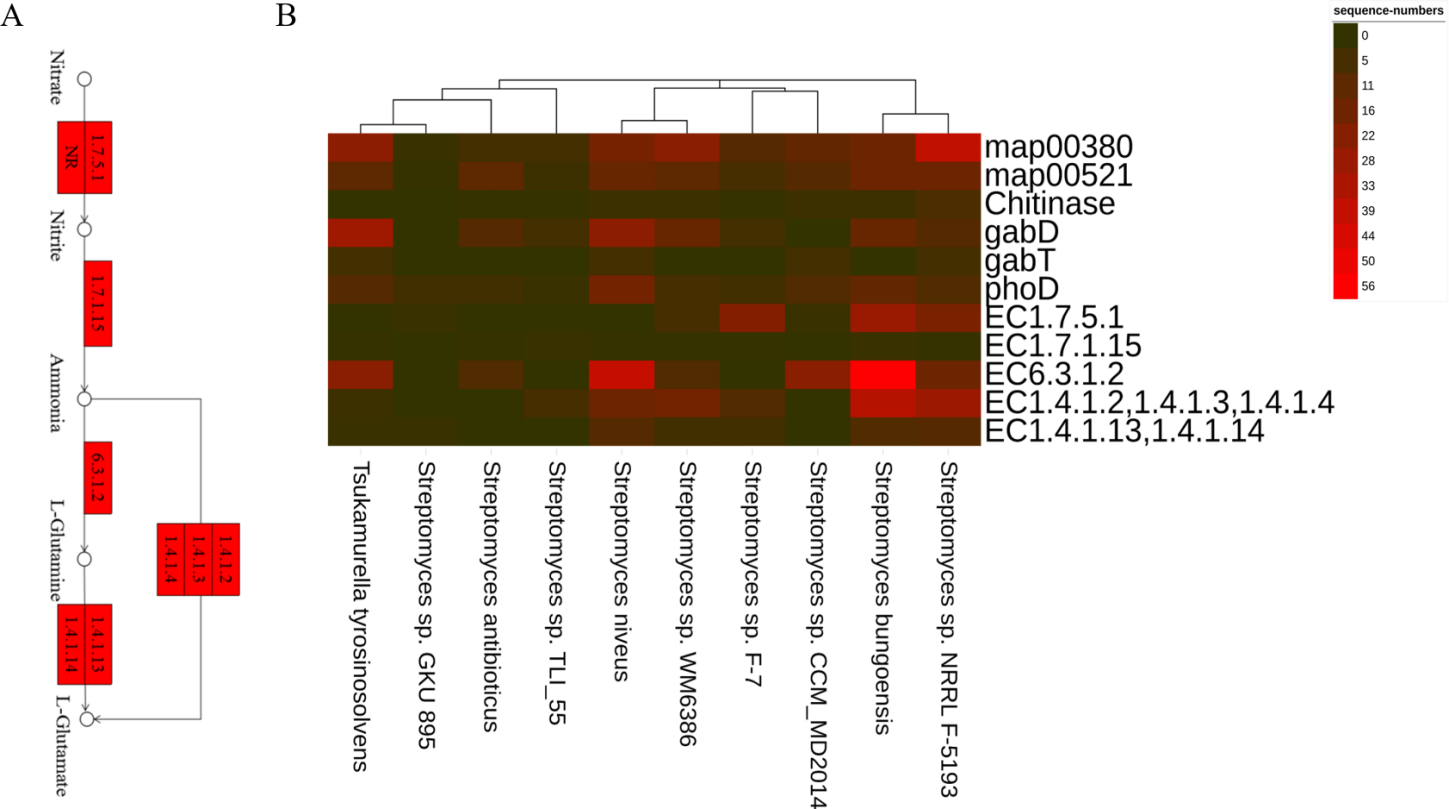
The PGPTs-related pathways, genes and enzymes annotated in the root-associated actinobacteria of *S. miltiorrhiza*. (A) Actinobacteria from the root-associated of *S. miltiorrhiza* are involved in the pathway of glutamine and glutamic acid biosynthesis. Numbers inside boxes with red backgrounds represent EC numbers of the enzymatic reaction. (B) The number of nucleotide sequences of related metabolic pathways, genes, and enzymes was annotated in top 10 species. map00380: Tryptophan metabolism. map00521: Streptomycin biosynthesis. *gabD* and *gabT* can encode GABA, GABA and Chitinase are related to antagonizing pathogens. *phoD*: encoding alkaline phosphatase. EC1.7.5.1: nitrate reductase in Nitrogen metabolism, catalyzes the reduction of nitrate to nitrite. EC1.7.1.15: nitrite reductase, which catalyzes nitrite to ammonia. EC6.3.1.2: glutamine synthetase, catalyzes ammonia to L-glutamine. EC1.4.1.2, 1.4.1.3, 1.4.1.4: glutamate dehydrogenase, catalyzes ammonia to L-glutamate. EC1.4.1.13,1.4.1.14: glutamate synthase, catalyzes L-glutamine to L-glutamate.

Besides, the genes and enzymes involved in plant growth-promoting traits (PGPTs) were also annotated (Figs. 4 and 5), including, enzymes related to IAA synthesis (EC1.13.12.3, EC3.5.1.4, EC1.4.3.4, EC1.2.1.3), genes involved in nitrogen metabolism (*nifU*, *narG*, *narI*, *nirB*, *nirD*, *nirK*, *nxrB*, *norB*, *glnA*, GLUL, *gltB*, *gltD*, *nxrA*, and *gdhA*), phosphorus metabolism (*phoD*, *phnG*, *phnI*, *phnJ* and *phnA*), and siderophore biosynthesis (*pchB*, *dhbF*, and *entF*). We have discovered the presence of chitinase and *β*-1,3-glucanase that help fight plant pathogens.

On the other hand, some antibiotic synthesis pathways have been annotated, including map00521(Streptomycin biosynthesis), map00524(Neomycin, kanamycin, and gentamicin biosynthesis). And some possible biosynthetic gene clusters involved in secondary metabolite production, such as NRPS, NRPS-like, T1PKS, and hgIE-KS, were also annotated using the AntiSMASH tool (Kai et al., 2019) (https://antismash.secondarymetabolites.org). The complete citric acid cycle was annotated in the root-associated actinobacteria, which can help microorganisms use insoluble inorganic phosphate salts (Fig. S1).

### Antimicrobial activity

**Figure 6:**
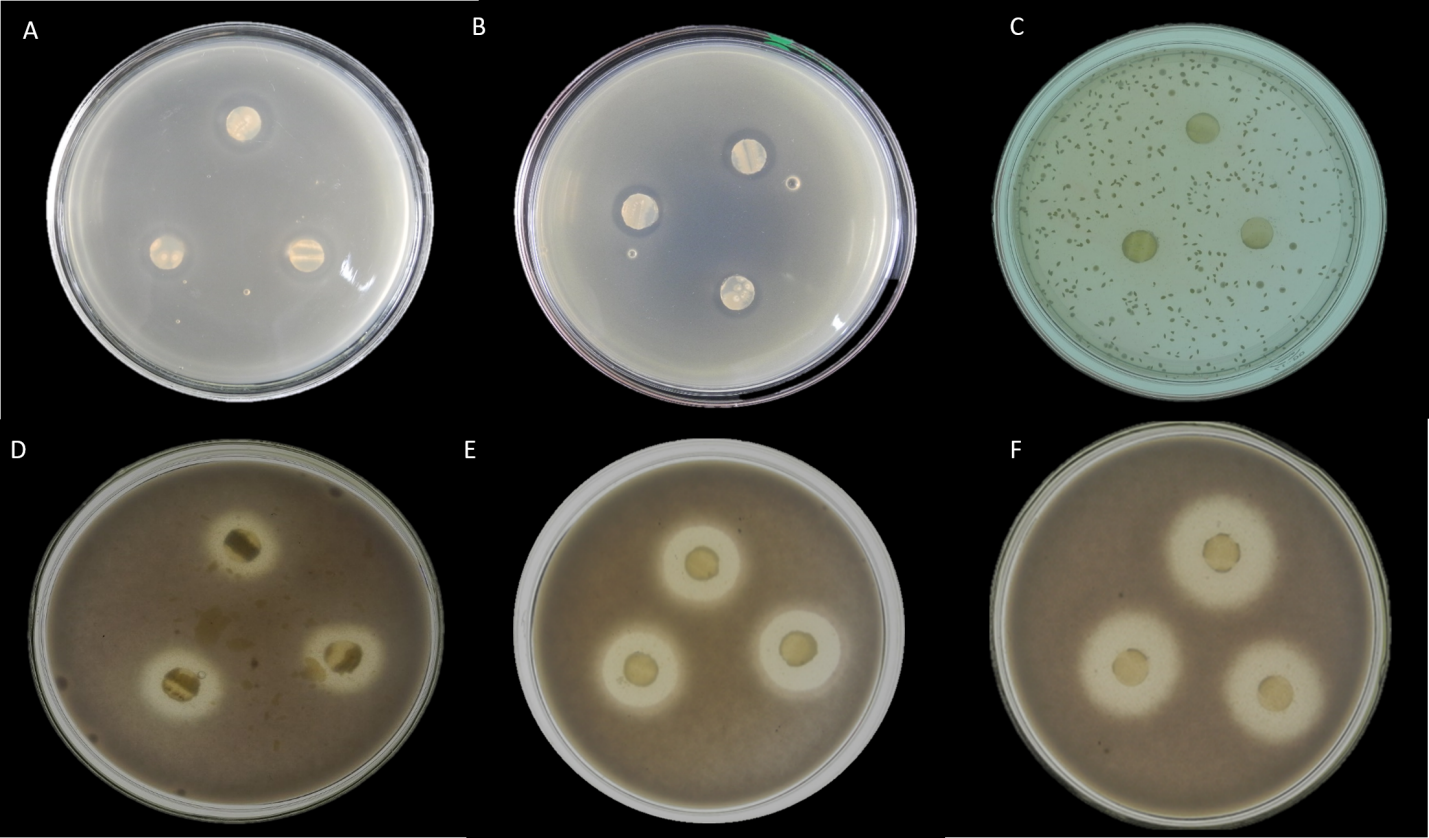
In vitro antibacterial activity of actinobacteria in the root-associated of *S. miltiorrhiza*. (A) Antagonistic effect of strain EA083 on *Escherichia coli*. (B) Antagonism of strain EA083 against *Staphylococcus aureus*. (C) Antagonistic effect of strain EA083 on *Candida albicans*. (D) Antagonistic effect of strain EA083 on *Fusarium oxysporum*. (E) Inhibition of *Fusarium oxysporum* by strain EA166. (F) Strain SA107 inhibition of *Fusarium oxysporum*.

Here, seventeen isolates exhibited antimicrobial activity against indicator microorganisms. Most of the active isolates showed antimicrobial activity against *Staphylococcus aureus* and *Fusarium oxysporum*. Among these tested isolates, thirteen strains were shown antimicrobial activity against *Staphylococcus aureus*, and eight strains were showed antimicrobial activity against *Fusarium oxysporum*. There were three strains and one strain that had inhibitory effects on *Candida albicans* and *Escherichia coli*, respectively. The EA083 strain (the similarity with *Streptomyces lavendulae* NRRL B-1230 is 99.71%) showed a high and broad antimicrobial spectrum (Fig. 6).

### Plant growth-promoting activity

**Figure 7:**
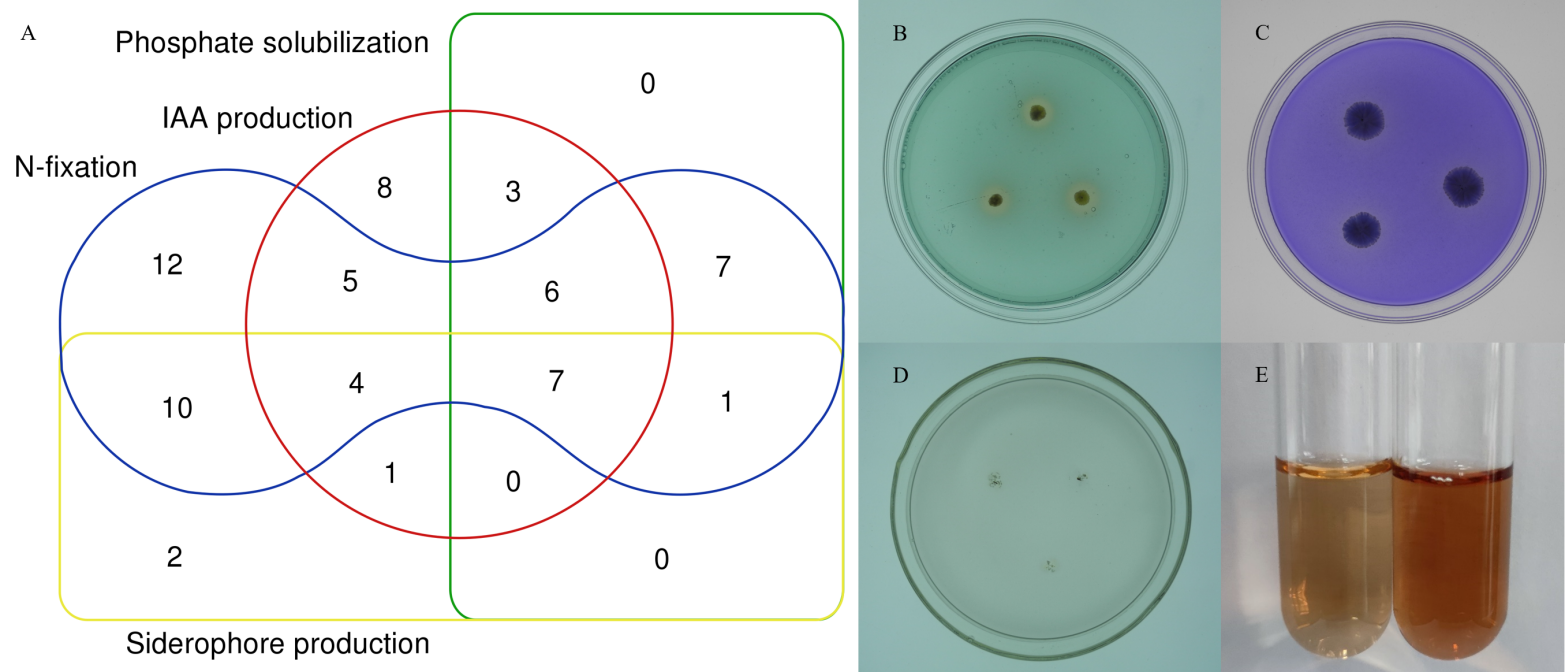
In vitro PGPTs activity of actinobacteria from the root-associated of *S. miltiorrhiza*. (A) Venn diagram representation of plant growth promoting actinobacterial isolates showing different PGP traits. (B) The yellow halo shows the siderophore activity of strain SA036. (C) The halo shows the phosphate solubilizing activity of strain SA036. (D) Strain SA036 can grow on nitrogen-free medium. (E) The left is the control and the red production on the right shows that the strain SA036 has the activity of producing IAA.

To further characterize the root-associated actinobacterial isolates, their PGPTs were tested. These isolates showed different PGP activities (Table S5). Of 72 strains, 24 isolates had phosphate solubilizing properties, 52 isolates grew on an N-free medium. 37 isolates (51.3%) produced IAA, with an average content of 2.48–21.62 µg/mL, and SA047 had the highest IAA content. Of the total 72 isolates, 25 isolates showed siderophore production. 7 isolates showed all tested PGPTs (Fig. 7).

## Discussion

### The species diversity of culturable root-associated actinobacteria of *S. miltiorrhiza*

Three hundred and sixty-nine root-associated actinobacterial of *S. miltiorrhiza* were isolated in this study, belonging to forty-six species in four genera, among which *Streptomyces* was the dominant genus. *Streptomyces* was widely present in various habitats. Many studies have shown that, in various plants, it is the dominant genus of endophytic and rhizosphere actinobacteria (Kim et al., 2012; Kaewkla & Franco, 2013; Dinesh et al., 2017; Bulgarelli et al., 2012). Li et al. (2010) and Yan et al. (2016) isolated *Streptomyces* from *S. miltiorrhiza*, and the same genus was obtained in this study. Moreover, different from previous studies, the genera *Tsukamurella* and *Nocardiopsis* were isolated. To our knowledge, this is the first study that reported *Tsukamurella* and *Nocardiopsis* isolation from the tissues of *S. miltiorrhiza*. Besides, the isolates of EA050, EA140, and SA079 shared low 16S rRNA gene sequence similarities (<98.65%, the threshold for differentiating two species) with validly described species based on the results of BLAST search in NCBI, indicating that these isolates might represent novel *Streptomyces* species (Kim et al., 2014). In this study, *Streptomyces turgidiscabies* and *Streptomyces acidiscabies* were isolated. They reportedly cause potato scabies (Santos-Cervantes et al., 2016). However, they did not cause tissue lesions *S. miltiorrhiza*, and the reason remains are unclear. We speculated that it might be related to the root-associated microbial system balance.

### The functional diversity of culturable root-associated actinobacteria of *S. miltiorrhiza*

Metagenomics help explore and discover the genes and functions of microorganisms in natural environments that are unreachable through classical microbial cultivation techniques. Although culture-independent approaches clarify potential microbial roles, culture-based studies are still important for practical microbial use. Some scholars have used metagenomics to conduct functional analysis of culturable microorganisms (Costa et al., 2020; Diaz-Garcia, Bugg & Jimenez, 2020). This implies we can combine the strengths of both metagenomics and cultivation techniques to understand the functions of culturable microorganisms. This study found that the root-associated actinobacteria of *S. miltiorrhiza* have rich functional genes, and have the potential to promote plant growth, protect plant against pathogens, and synthesize a variety of biologically active ingredients.

### Plant growth promotion

Actinobacteria can promote plant growth by making nutrients/substrates (e.g., phosphorous, nitrogen, and iron) accessible to the host plant and producing various plant hormones (Liu et al., 2017; Purushotham et al., 2018; Borah & Thakur, 2020). IAA is an important plant hormone beneficial to plant growth. Many studies have reported that *Streptomyces* could produce IAA, promoting plant growth and increasing seed germination rate (Dochhil, Dkhar & Barman, 2013; Lin & Xu, 2013; Myo et al., 2019). Nitrogen and phosphorus are indispensable nutrients for plant growth and development. There is evidence that the root-associated actinobacteria can increase plant nutrient utilization by fixing nitrogen and dissolving phosphorus, consequently promoting plant growth (Anita & Richau, 2013; Solans et al., 2019). Iron is an essential trace mineral for almost all organisms, and it influences various activities and cell metabolisms, such as respiration and photosynthesis. However, iron availability is often limited due to its poor solubility (Cao et al., 2020). Studies have found that some microorganisms can synthesize siderophores to help plants acquire iron in the soil (Crowley et al., 1991). Besides, they can also compete with pathogens for iron to produce antagonism, which is also an important mechanism for antagonizing pathogens (Kamil et al., 2018). As far as we know, among the 46 species of actinobacteria in this study, many species reportedly promote plant growth. For example, *Nocardiopsis dassonoillei* and *Streptomyces violaceoruber* can promote wheat and pepper seedling growth, respectively (Sang et al., 2013; Allali et al., 2019).

In our study, the enzymes related to IAA synthesis (EC1.13.12.3, EC3.5.1.4, EC1.4.3.4, EC1.2.1.3) and genes related to nitrogen fixation (*nifU*), nitrification (*nxrB*), denitrification (*nirK*, *norB*) were annotated. Moreover, genes coding alkaline phosphatase(*phoD*), C-P lyase (*phnG*, *phnI*, and *phnJ*), C-P hydrolase (*phnA*), and siderophores biosynthesis (*pchB*, *dhbF*, and *entF*), were also found. In particular, we found that the above-mentioned related genes and enzymes are abundant in the top ten abundance species (Fig. 5). Besides, in vitro experiments proved the correctness of function prediction, and 37 strains produced IAA in the presence of tryptophan, 52 isolates had nitrogen-fixing characteristics, and 24 strains had phosphorus-dissolving characteristics, and 25 strains had siderophores-producing characteristics. In summary, it is indicated that the root-associated actinobacteria of *S. miltiorrhiza* might participate in IAA biosynthesis, siderophores production, and nitrogen and phosphorus metabolism in plants and soil by producing associated enzymes.

### Antagonists pathogens

Disease is the main biotic stress factor leading to crop productivity reduction, and *S. miltiorrhiza* is no exception. The incidence of root rot caused by *Fusarium oxysporum* on *S. miltiorrhiza* roots is high in China. It has been earlier reported that the root-associated actinobacteria could effectively inhibit plant pathogens, thus protecting the host plants from diseases (Al-Askar et al., 2015; Muangham, Pathom-aree & Duangmal, 2015; Soltanzadeh, Nejad & Bonjar, 2016). Several antibiotic synthesis pathways were annotated in this study, including map00521(Streptomycin biosynthesis), map00524(Neomycin, kanamycin, and gentamicin biosynthesis). Moreover, phenazine (map00405) and chitinase (EC3.2.1.14) biosynthetic genes/pathways have been noted. Antibiotics, phenazines, and chitinase reportedly have an antagonistic effect on plant pathogens (Singh & Gaur, 2016; Aamir et al., 2020). Actinobacteria, with the activity of synthesizing antibiotics and chitinase, are used as biological control agents against plant pathogens (Al-Askar et al., 2015; Abbasi et al., 2019). Furthermore, our study also revealed the presence of *gab* D and *gab* T known to be significant genes during *γ*-aminobutyric acid (GABA) production, which helps suppress plant pests and diseases (Belbahri et al., 2017). Simultaneously, of the 46 species isolated, many have been reported to exhibit antibiotic synthesis, chitin degradation, and plant pathogen antagonism, such as *Tsukamurella tyrosinosolvens* could produce antagonistic effects against *Phytophthora Capsici* (Sang et al., 2013).

Therefore, we speculate that the root-associated actinobacteria of *S. miltiorrhiza* exhibited plant-pathogens antagonism. Fortunately, antimicrobial experiments confirm our conjecture. Eight strains inhibited *Fusarium oxysporum*; among them, the EA083, EA166, and SA107 strains showed noticeable inhibitory effects (Fig. 6). This result shows that the root-associated actinobacteria might play a key role in *Fusarium oxysporum* antagonism by producing antimicrobial compounds and chitinase. In summary, the root-associated actinobacteria of *S. miltiorrhiza* have great potential as a biological control agent. Also, we found that the root-associated actinobacteria of *S. miltiorrhiza* exhibited inhibitory effects on human pathogenic bacteria, suggesting that it can be used in the agricultural and pharmaceutical industries.

### Biosynthetic potential of isolates

Actinobacteria have always been one of the primary sources of bioactive ingredient production. As expected, we found that the culturable root-associated actinobacteria of *S. miltiorrhiza* have the potential to synthesize bioactive ingredients. Studies have reported that active metabolite synthesis was evaluated using specific primer pairs that target PKS-I, II, and NRPS (Katz & Baltz, 2016; Peng et al., 2020). In our study, we identified possible biosynthetic gene clusters involved in secondary metabolite production. We also found that related pathways (map01052 and map01056) were annotated. Besides, our study also noted the biosynthetic pathways of flavones (map00944, map00941, and map000943), and alkaloids (map00960, map00950). It is indicated that the root-associated actinobacteria *S. miltiorrhiza*. have the potential to synthesize different bioactive ingredients.

Surprisingly, we also found that it has the activity of degrading aromatic compounds, including polycyclic aromatic hydrocarbons, toluene, xylene, which are remarkably harmful organic pollutants in the soil (Peng et al., 2020). Compared to physical and chemical technology, the plant-related microbe can excellently remediate aromatic compound pollutants from contaminated sites due to its environmentally friendly advantages. In this study, the abundance of ko involved in degrading aromatic compounds was annotated. The average relative abundance of ko00362 (Benzoate degradation), ko00627 (Aminobenzoate degradation), and ko00625 (Chloroalkane and chloroalkene degradation) were greater than 0.1%. It is shown that the culturable root-associated actinobacteria of *S. miltiorrhiza*, as soil amendments, can degrade aromatic compounds.

## Conclusions

This study showed the diversity and function of the culturable root-associated actinobacterial communities of *S. miltiorrhiza* in Sichuan, China. To our knowledge, this is the first study to report the functional analysis of the culturable root-associated actinobacteria of *S. miltiorrhiza* by metagenomics. A total of 369 culturable actinobacterial strains were isolated and affiliated to four genera, among which *Streptomyces* was the dominant genus. Metagenomic data revealed that the root-associated actinobacteria possess genes and enzymes involved in IAA synthesis, siderophores, nitrogen and phosphorus metabolism, and plant pathogen antagonism. Simultaneously, in vitro studies proved the reliability of the metagenomics results. The results showed that the root-associated actinobacteria exhibit rich in diversity and function and might play an important role in the growth and health of the plant, which have great potential as plant growth promoters and biological control agents. However, in vitro assays are ultimately limited, and the details about how strains influence the plant need to be confirm by the greenhouse experiment. This study provides new insights into understanding the function of the culturable root-associated actinobacteria and provides a basis to further study the interaction of actinobacteria and host plants in the roots of *S. miltiorrhiza*.

##  Supplemental Information

10.7717/peerj.11749/supp-1 Supplemental Information 1 Closest BLASTN matches for the 16S rDNA sequence and antimicrobial activity of the antimicrobial isolates “-” means No inhibition, “+” means the diameter of inhibition zone in the range of 10-15 mm, “++” means the diameter of inhibition zone in the range of 15–20 mm, “+++” means the diameter of inhibition zone in the range of 20–25 mm. “*S.*” *Streptomyces*, “*N.*” means *Nocardiopsis*, “*T.*” means *Tsukamurella*, and “*K.*” means *Kitasatospora*. B1 means *Escherichia coli*, B2 means *Staphylococcus aureus*, B3 means *Bacillus cereus*, B4 means *Pseudomonas aeruginosa*, F1 means *Candida albicans*, and F2 means *Fusarium oxysporum*.

10.7717/peerj.11749/supp-2 Supplemental Information 2 The metabolism pathway with a relative abundance greater than 0.1% in the annotated by the KEGG database

10.7717/peerj.11749/supp-3 Supplemental Information 3 The number of genes annotated to level 1 of the CAZy database

10.7717/peerj.11749/supp-4 Supplemental Information 4 The number of genes annotated to level 1 of the eggNOG database

10.7717/peerj.11749/supp-5 Supplemental Information 5 Screening of the root-associated actinobacterial isolates for *in vitro* plant growth promoting traits “-” means negative; “+” positive.

10.7717/peerj.11749/supp-6 Supplemental Information 6 Citrate cycle (TCA cycle) was annotated in KEGG Photo credit: KEGG database.

## Additional Information and Declarations

### DNA Deposition

The following information was supplied regarding the deposition of DNA sequences: The group sequences are available at GenBank: MW642091 to MW642162.

### Data Availability

The following information was supplied regarding data availability: The raw measurements are available in the Supplemental Files.
