# Supplementary material for: Diversity and function of culturable actinobacteria in the root-associated of Salvia miltiorrhiza Bunge
Source: PeerJ. 2021 Jul 9;9:e11749. doi: 10.7717/peerj.11749 (PMC8274492; doi:10.7717/peerj.11749)
Supplement: Supplemental Information 1 — “-” means No inhibition, “+” means the diameter of inhibition zone in the range of 10-15 mm, “++” means the diameter of inhibition zone in the range of 15–20 mm, “+++” means the diameter of inhibition zone in the range of 20–25 mm. “S.” Streptomyces, “N.” means Nocardiopsis, “T.” means Tsukamurella, and “K.” means Kitasatospora. B1 means Escherichia coli, B2 means Staphylococcus aureus, B3 means Bacillus cereus, B4 means Pseudomonas aeruginosa, F1 means Candida albicans, and F2 means Fusarium oxysporum. [file peerj-09-11749-s001.docx]

| isolation | B1 | B2 | B3 | B4 | F1 | F2 | Closest cultivated species (GenBank accession no.) | Similarity (%) |
| --- | --- | --- | --- | --- | --- | --- | --- | --- |
| EA006 | - | - | - | - | - | - | *S. canus* NBRC 12872 (NR_041085.1) | 99.55 |
| EA007 | - | - | - | - | - | - | *S.* *osmaniensis* (LC136922.1) | 99.40 |
| EA009 | - | - | - | - | - | - | *S.* *thermocarboxydus* NBRC 16323 (AB249926.1) | 99.85 |
| EA012 | - | - | - | - | - | - | *S.* *polychromogenes* subsp. arenicolus NBRC 13872 (AB184541.1) | 99.93 |
| EA021 | - | - | - | - | - | - | *S.* *fimicarius* NBRC 13037 (NR_112347.1) | 100 |
| EA024 | - | **+** | - | - | - | - | *S.* *dioscori* (MF100125.1) | 99.48 |
| EA039 | - | **+** | - | - | - | - | *S. canus* NBRC 12872 (NR_041085.1) | 99.71 |
| EA040 | - | - | - | - | - | - | *S.* *glaucescens* DSM 40716 (X79322.1) | 99.12 |
| EA043 | - | **+** | - | - | - | - | *S.* *albogriseolus* NBRC 3709 (NR_112489.1) | 99.71 |
| EA050 | - | - | - | - | - | - | *S.* *lacrimifuminis* (MH182607.1) | 98.22 |
| EA060 | - | - | - | - | - | - | *S.* *griseorubens* AS 4.1839 (AY999870.1) | 99.63 |
| EA062 | - | - | - | - | - | - | *S.* *macrosporeus* NBRC 13897 (AB184542.1) | 100 |
| EA071 | - | - | - | - | - | - | *N.* *dassonoillei* DSM 43111 (NR_074635.1) | 99.85 |
| EA074 | - | - | - | - | - | - | *S.* *tempisquensis* (KF954543.1) | 99.85 |
| EA077 | - | - | - | - | - | **+** | *S.* *sampsonii* DSM 40394T (Z76680.1) | 99.85 |
| EA078 | - | - | - | - | - | - | *S. griseorubens* AS 4.1839 (AY999870.1) | 99.50 |
| EA080 | - | **+** | - | - | - | - | *S.* *camponoticapitis* (LC551874.1) | 99.92 |
| EA082 | - | - | - | - | - | - | *S.* *atriruber* NRRL B-24165 (EU812169.1) | 99.12 |
| EA083 | **+** | **+** | - | - | **+++** | **++** | *S.* *lavendulae* NRRL B-1230 (NR_117992.2) | 99.71 |
| EA090 | - | - | - | - | - | - | *S. canus* NBRC 12872 (NR_041085.1) | 99.48 |
| EA098 | - | - | - | - | - | - | *S. gancidicus* NRRL B-1872 (NR_115459.1) | 99.23 |
| EA102 | - | - | - | - | - | - | *S. rubrogriseus* DSM 41477(NR_114667.1) | 99.71 |
| EA107 | - | - | - | - | - | - | *S. rubrogriseus* DSM 41477 (NR_114667.1) | 99.71 |
| EA108 | - | **+** | - | - | - | **+** | *S. enissocaesillis* NRRL B-16365 (AB915613.2) | 98.81 |
| EA114 | - | - | - | - | - | - | *S. fimicarius* NBRC 13037 (NR_112347.1) | 99.85 |
| EA123 | - | - | - | - | - | - | *S. rubrogriseus* DSM 41477 (NR_114667.1) | 99.85 |
| EA127 | - | - | - | - | - | - | *S. acidiscabies* ATCC 49003 (FJ007426.1) | 100 |
| EA128 | - | - | - | - | - | - | *S. violaceoruber* DSM 40049 (NR_041914.1) | 99.85 |
| EA138 | - | - | - | - | - | - | *S. coelicoflavus* JCM 6918 (AY999752.1) | 100 |
| EA140 | - | - | - | - | - | - | *S. lacrimifuminis* (MH182607.1) | 98.57 |
| EA148 | - | - | - | - | - | - | *S. hokutonensis* (AB808756.1) | 99.64 |
| EA159 | - | - | - | - | **++** | **+** | *S. paulus* NBRC 14877 (AB184626.1 ) | 100 |
| EA166 | - | - | - | - | **+++** | **++** | *S. plumbeus* NBRC 13708 (AB184468.2) | 99.48 |
| EA169 | - | - | - | - | - | - | *S. violaceoruber* DSM 40049 (NR_041914.1) | 99.85 |
| EA173 | - | - | - | - | - | - | *S. exfoliatus* AS 4.1874 (AY999898.1) | 99.34 |
| EA174 | - | - | - | - | - | - | *S. fradiae* NBRC 12215 (AB184069.1) | 99.93 |
| EA179 | - | - | - | - | - | - | *S. canus* NBRC 12872 (NR_041085.1) | 99.34 |
| EA180 | - | - | - | - | - | - | *S. peruviensis* DSM 40592 (AJ310924.1) | 99.27 |
| EA181 | - | - | - | - | - | - | *S. canus* NBRC 12872 (NR_041085.1) | 99.63 |
| EA182 | - | - | - | - | - | - | *S. pseudovenezuelae* ISP 5212 (AJ399481.1) | 99.48 |
| EA183 | - | - | - | - | - | - | *T. tyrosinosolvens* JCM 15482 (KX924546.1) | 100 |
| EA186 | - | - | - | - | - | - | *S. marokkonensis* LMG 23016 (NR_114960.1) | 99.19 |
| SA005 | - | **++** | - | - | - | - | *S. cacaoi* subsp. asoensis NBRC 13813 (NR_112416.1) | 100 |
| SA010 | - | - | - | - | - | - | *S. canus* NBRC 12872 (NR_041085.1) | 99.42 |
| SA014 | - | - | - | - | - | - | *S. argillaceus* (MF462922.1) | 99.63 |
| SA015 | - | + | - | - | - | - | *S. osmaniensis* (LC136922.1) | 99.34 |
| SA019 | - | - | - | - | - | - | *S. gancidicus* NRRL B-1872 (NR_115459.1) | 99.06 |
| SA023 | - | **+** | - | - | - | - | *S. rishiriensi* NBRC 13407 (AB184383.1) | 99.55 |
| SA033 | - | - | - | - | - | - | *S. aquilus* (MH718844.1) | 99.78 |
| SA035 | - | - | - | - | - | - | *S. bungoensis* NBRC 15711 (AB184696.1) | 99.56 |
| SA036 | - | - | - | - | - | - | *K. purpeofusca* LMG 20283 (NR_042304.1) | 99.26 |
| SA037 | - | - | - | - | - | - | *S. glaucescens* DSM40716 (X79322.1) | 99.19 |
| SA046 | - | - | - | - | - | - | *S. bungoensis* NBRC 15711 (AB184696.1) | 99.78 |
| SA047 | - | **+** | - | - | - | **+** | *S. tauricus* NRRL B-12497 (NR_116383.1) | 100 |
| SA050 | - | - | - | - | - | - | *S. lannensis* (KF703720.1) | 99.35 |
| SA060 | - | - | - | - | - | - | *S. longisporoflavus* NBRC 12886 (NR_112320.1) | 99.63 |
| SA064 | - | - | - | - | - | - | *S. fradiae* NBRC 12215 (AB184069.1) | 99.55 |
| SA065 | - | - | - | - | - | - | *S. fradiae* NBRC 12215 (AB184069.1) | 99.63 |
| SA067 | - | **+** | - | - | - | - | *S. pseudovenezuelae* ISP 5212 (AJ399481.1 ) | 99.56 |
| SA074 | - | - | - | - | - | - | *S. clavifer* NRRL B-2557 (DQ026670.1) | 100 |
| SA075 | - | **+** | - | - | - | - | *S. rishiriensi* NBRC 13407 (AB184383.1) | 99.78 |
| SA076 | - | **-** | - | - | - | **+** | *S. turgidiscabies* NBRC 16080 (NR_ 112583.1) | 99.63 |
| SA079 | - | - | - | - | - | - | *S. lacrimifluminis* (MH182607.1) | 98.51 |
| SA085 | - | - | - | - | - | - | *S. marokkonensis* LMG 23016 (NR_114960.1) | 99.12 |
| SA089 | - | - | - | - | - | - | *S. fimicarius* NBRC 13037 (NR_112347.1) | 99.78 |
| SA098 | - | - | - | - | - | - | *S. glaucescens* DSM40716 (X79322.1) | 99.18 |
| SA107 | - | **+** | - | - | - | **++** | *S. rapamycinicus* ATCC 29253 (NR_044199.1) | 98.91 |
| SA113 | - | - | - | - | - | - | *S. camponoticapitis* (LC551874.1) | 99.85 |
| SA118 | - | - | - | - | - | - | *S. hokutonensis* (AB808756.1) | 99.85 |
| SA122 | - | - | - | - | - | - | *S. longisporus* NBRC 12885 (NR_112319.1) | 99.85 |
| SA128 | - | - | - | - | - | - | *S. albogriseolus* NBRC 3709 (NR_112489.1) | 99.93 |
| SA136 | - | - | - | - | - | - | *S. rhizophilus* (MH432656.1) | 98.81 |
