# Supplementary material for: Diversity and function of culturable actinobacteria in the root-associated of Salvia miltiorrhiza Bunge
Source: PeerJ. 2021 Jul 9;9:e11749. doi: 10.7717/peerj.11749 (PMC8274492; doi:10.7717/peerj.11749)
Supplement: Supplemental Information 5 — “-” means negative; “+” positive. [file peerj-09-11749-s005.docx]

| isolation | IAA production  (µg/mL) | Siderophore production | Nitrogen fixation | Phosphate solubilization |
| --- | --- | --- | --- | --- |
| EA006 | 10.32 | - | + | + |
| EA007 | 8.62 | + | + | + |
| EA009 | 7.38 | - | - | - |
| EA012 | - | - | + | + |
| EA021 | - | - | + | - |
| EA024 | - | + | + | - |
| EA039 | 13.62 | - | + | - |
| EA040 | 10.42 | - | - | - |
| EA043 | 3.07 | - | + | - |
| EA050 | - | - | + | + |
| EA060 | - | + | + | - |
| EA062 | 13.58 | + | + | - |
| EA071 | 6.38 | + | + | + |
| EA074 | - | + | + | - |
| EA077 | 2.48 | + | + | - |
| EA078 | - | - | + | - |
| EA080 | - | - | + | - |
| EA082 | 10.63 | + | - | - |
| EA083 | 13.43 | + | + | + |
| EA090 | 12.31 | + | + | + |
| EA098 | 10.95 | - | - | - |
| EA102 | - | + | + | - |
| EA107 | - | + | + | - |
| EA108 | 3.10 | + | + | - |
| EA114 | 8.46 | - | + | - |
| EA123 | - | - | + | + |
| EA127 | - | - | + | + |
| EA128 | - | - | + | - |
| EA138 | - | - | + | - |
| EA140 | - | - | + | + |
| EA148 | - | - | + | + |
| EA159 | 8.03 | - | + | - |
| EA166 | 11.86 | - | - | - |
| EA169 | 3.05 | + | + | - |
| EA173 | - | + | + | - |
| EA174 | - | + | - | - |
| EA179 | 6.04 | - | + | + |
| EA180 | - | + | - | - |
| EA181 | 8.43 | - | + | - |
| EA182 | 16.78 | + | + | + |
| EA183 | 7.67 | + | + | - |
| EA186 | - | - | - | - |
| SA005 | 12.91 | - | - | + |
| SA010 | 7.98 | - | + | + |
| SA014 | 14.30 | - | + | - |
| SA015 | 14.25 | + | + | + |
| SA019 | 4.60 | - | + | + |
| SA023 | 19.89 | - | - | + |
| SA033 | 13.65 | - | + | + |
| SA035 | 10.25 | - | - | - |
| SA036 | 15.44 | + | + | + |
| SA037 | 12.59 | - | - | - |
| SA046 | - | - | - | - |
| SA047 | 21.62 | - | + | + |
| SA050 | 8.62 | + | + | - |
| SA060 | - | + | + | - |
| SA064 | - | + | + | + |
| SA065 | - | - | + | + |
| SA067 | - | - | - | - |
| SA074 | - | + | + | - |
| SA075 | - | - | - | - |
| SA076 | - | - | + | - |
| SA079 | - | - | + | - |
| SA085 | - | - | + | - |
| SA089 | - | - | + | - |
| SA098 | 10.88 | - | - | + |
| SA107 | - | - | - | - |
| SA113 | - | - | + | - |
| SA118 | - | - | + | - |
| SA122 | 7.64 | - | - | - |
| SA128 | - | - | - | - |
| SA136 | 17.04 | - | - | - |
