# Supplementary figures and images for: Diversity and function of culturable actinobacteria in the root-associated of Salvia miltiorrhiza Bunge
Source: PeerJ. 2021 Jul 9;9:e11749. doi: 10.7717/peerj.11749 (PMC8274492; doi:10.7717/peerj.11749)

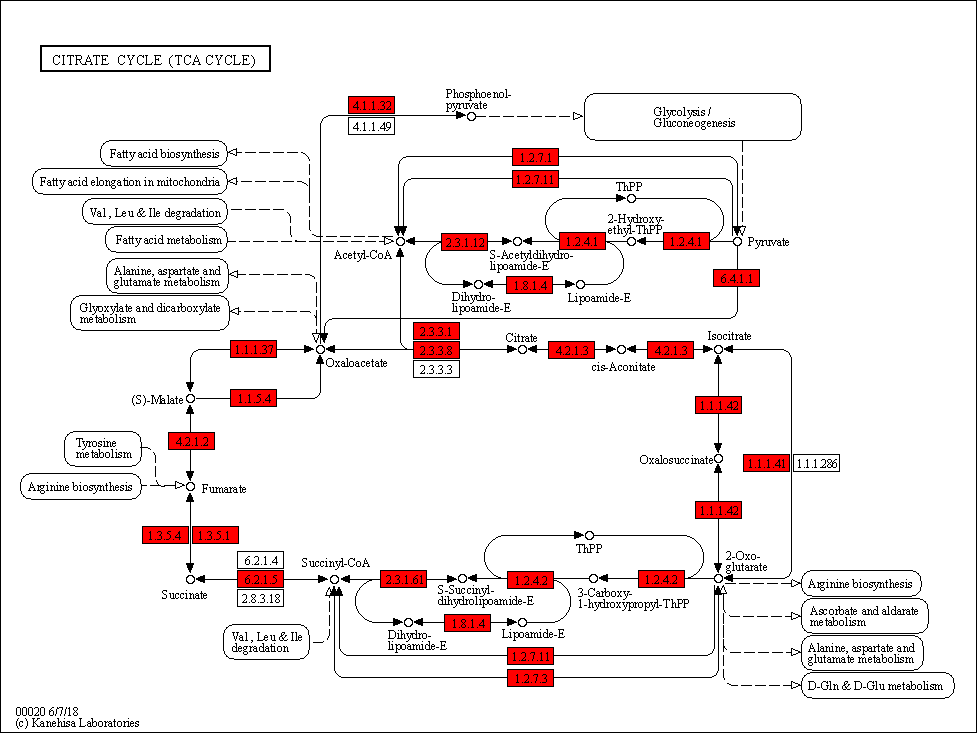

Supplement: Supplemental Information 6 — Photo credit: KEGG database. [file peerj-09-11749-s006.png]
